# Are clinical outcomes affected by laminoplasty method and K-line in patients with cervical ossification of posterior longitudinal ligament? A multicenter study

**DOI:** 10.1186/s13018-022-03407-8

**Published:** 2022-11-24

**Authors:** Nan Li, Sai Ma, Fangfang Duan, Yi Wei, Da He, Narihito Nagoshi, Kota Watanabe, Masaya Nakamura, Morio Matsumoto, Hyeongseok Jeon, J. J. Lee, Keung-Nyun Kim, Yoon Ha, Kenny Kwan, A. K. P. Cheung, Aaron Clark

**Affiliations:** 1 Department of Spine Surgery, Beijing Jishuitan Hospital, The Fourth Medical College of Peking University, 31 Xinjiekou Dongjie, Xicheng District, Beijing, 100035 China; 2 Department of Orthopaedic Surgery, Keio University School of Medicine, Tokyo, Japan; 3 Department of Neurosurgery, Spine and Spinal Cord Institute, Yonsei University College of Medicine, Seoul, Republic of Korea; 4 Department of Neurosurgery, Kangbuk Samsung Hospital, Sungkyunkwan University College of Medicine, Seoul, Republic of Korea; 5 POSTECH Biotech Center, Pohang University of Science and Technology (POSTECH), Pohang, Gyeongbuk 37673 Republic of Korea; 6 Department of Orthopaedics and Traumatology, LKS Faculty of Medicine, The University of Hong Kong, Hong Kong, China; 7 Department of Neurological Surgery, University of California, San Francisco, CA USA

**Keywords:** French-door, K-line, Laminoplasty, Open-door, Ossification of the posterior longitudinal ligament

## Abstract

**Background:**

Open-door laminoplasty (ODL) and French-door laminoplasty (FDL) are the main laminoplasty techniques used to treat cervical ossification of the posterior longitudinal ligament (C-OPLL). However, few studies have compared the outcomes of ODL and modified FDL (mFDL) for C-OPLL. We explored the differences in outcomes between ODL and mFDL for C-OPLL and analyzed the technical efficacy of each procedure in patients with K-line (+) or (−) C-OPLL.

**Methods:**

From January 2010 to December 2015, 202 patients with K-line (+) or (−) C-OPLL were retrospectively recruited from 4 institutions. Clinical outcomes were evaluated using the Japanese Orthopaedic Association (JOA) score, JOA score recovery rate, operative time, blood loss, and complications. Univariate analysis and binary logistic regression models were adjusted for confounding factors.

**Results:**

Two hundred patients (mFDL, *n* = 69; ODL, *n* = 131) with a median follow-up of 42 months (range 36–54 months) were included. The postoperative JOA score significantly improved in both groups (*P* < 0.05). After adjusting for confounding factors, there was a statistically significant difference in blood loss (≥ 300 mL) between the two groups (*P* = 0.005), but there was no significant difference in the postoperative JOA score (≥ 14) (*P* = 0.062), JOA score recovery rate (≥ 0.82) (*P* = 0.187), or operative time (≥ 90 min) (*P* = 0.925). C5 palsy tended to occur more often in the mFDL group, although the difference was not significant (*P* > 0.05). The stratified analysis of the K-line status showed more blood loss in K-line (+) patients who underwent mFDL, but there was no significant difference in the postoperative JOA score, JOA score recovery rate, or operative time between the ODL and mFDL groups. Additionally, there was no significant difference in blood loss, postoperative JOA score, JOA score recovery rate, or operative time among all patients with K-line (+) or (−) C-OPLL in both groups.

**Conclusions:**

Both ODL and mFDL are effective for patients with C-OPLL. However, more blood loss tends to occur during mFDL. This study showed no significant difference in the operative time or incidence of complications between the two techniques. The efficacy of ODL and mFDL was not affected by the K-line status (+ or −) in patients with C-OPLL.

## Background

Cervical ossification of the posterior longitudinal ligament (C-OPLL) may narrow the cervical canal and cause myelopathy or radiculopathy or increase the risk of spinal cord injury following minor trauma [1, 2]. Laminoplasty is often indicated for patients with OPLL-induced cervical myelopathy because it can provide long-segment decompression and satisfactory preservation of the range of motion. Laminoplasty is usually classified as either open-door laminoplasty (ODL) or French-door laminoplasty (FDL) according to the decompressive method used. ODL was developed by Hirabayashi et al. [3], and FDL was developed by Kurokawa et al. [4] in the early 1980s, and several modifications were thereafter applied in the clinical setting. However, the potential differences in outcomes between these two techniques have seldom been considered. Which laminoplasty technique has higher effectiveness for patients with C-OPLL remains unclear [5, 6]. Moreover, although the outcome of laminoplasty for patients with K-line (+) C-OPLL is satisfactory [7], whether laminoplasty is appropriate for K-line (−) C-OPLL is unknown. Theoretically, the posterior shift of the spinal cord tends to be hindered in patients with thick ossification foci and/or kyphotic alignment [8–10]. However, few reports have focused on comparing the outcomes of the two laminoplasty techniques between patients with K-line (+) and (−) C-OPLL.

To fill this knowledge gap, we performed a retrospective study among four institutions in Asia. Our goal was to compare the clinical outcomes of two laminoplasty techniques for C-OPLL and determine whether there is a difference in effectiveness between patients with K-line (+) and (−) C-OPLL treated by ODL or FDL.

## Methods

### Patients

In total, 202 patients with C-OPLL were treated with ODL or modified FDL (mFDL) in 4 institutions from January 2010 to December 2015. mFDL was performed in the first author’s institution, and ODL was conducted in the other three institutions. All investigators were well-educated, experienced orthopedic spine surgeons or neurosurgeons; all had more than 15 years of clinical experience and had performed at least 150 operations for treatment of C-OPLL. The inclusion criterion was a cervical spinal disorder due to C-OPLL as demonstrated by computed tomography. The patients’ cervical myelopathy was confirmed with magnetic resonance imaging. Neurological dysfunction was detected by the following physical examinations: positive tandem gait or Romberg sign, spasticity, hyperreflexia, or positive pathologic reflex. The exclusion criteria were a history of cervical surgery, trauma, tumors, hormonal therapy, or infection. Medical documents, imaging features, and clinical outcomes were assessed preoperatively and postoperatively. The follow-up duration was ≥ 36 months. This study was designed in conformity with the Declaration of Helsinki.

### Surgical procedures

**Fig. 1:**
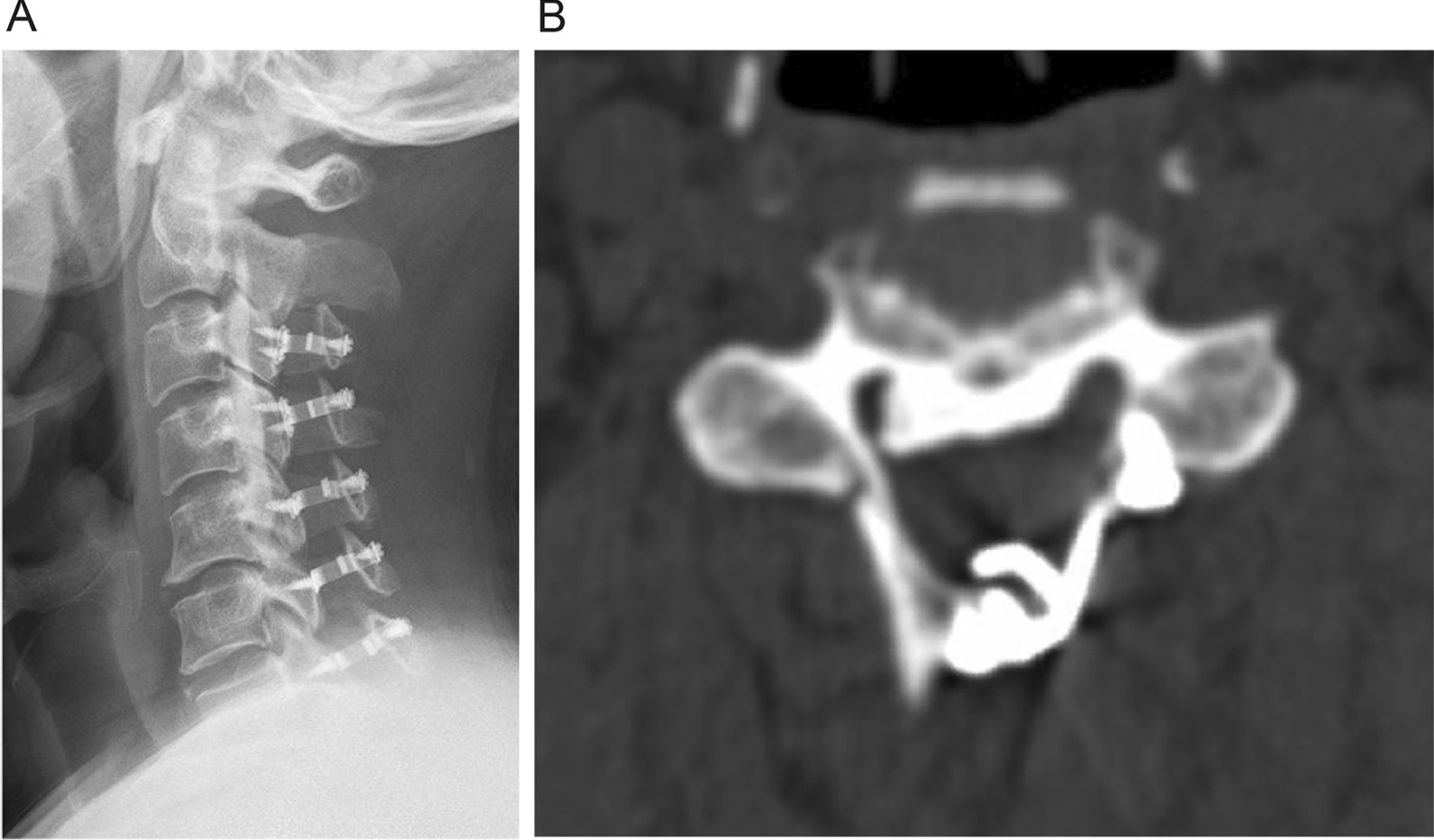
**A** and **B** Lateral X-ray and axial computed tomography slice of patients with C-OPLL undergoing ODL. The laminas were opened from one side, and the gaps were fixed with arch plates (C3–7)

ODL was performed according to the approach established by Hirabayashi et al. [3]. A midline incision was made along the spinous process, and the paravertebral muscles were then detached from the spinous processes and laminae. After removing the spinous processes, gutters were created on the bilateral laminae using a high-speed drill along the border between the laminae and facets. In patients with mild OPLL, the gutter on one side was prepared for a hinge; in patients with severe OPLL, the contralateral gutter was completely opened. Unilateral titanium mini-plates were applied to keep the laminae open (Centerpiece; Medtronic Sofamor Danek, Memphis, TN, USA) (Fig. 1A, B).

**Fig. 2:**
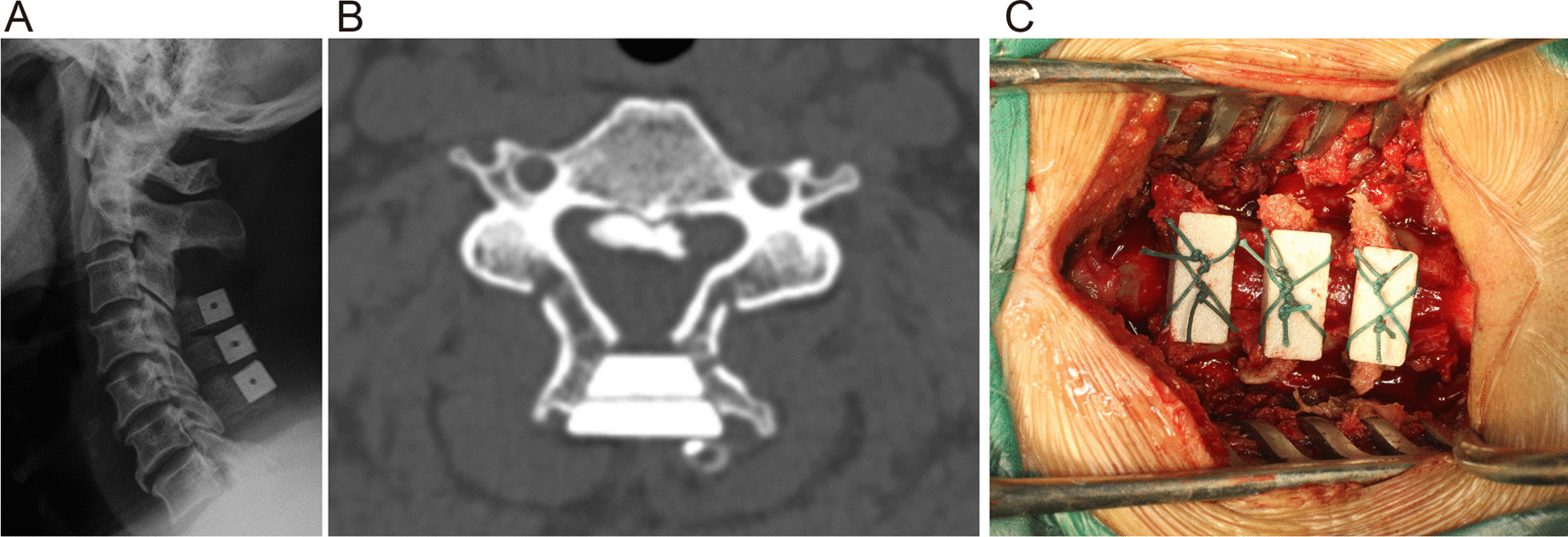
Lateral X-ray and axial computed tomography slice of patients with C-OPLL undergoing mFDL. **A** and **B** The laminas were opened from both sides, and the split spinous processes were fused with coralline hydroxyapatite (CHA) spacers. **C** Operative picture of mFDL, including laminectomy of C3 and the upper half of C7 as well as fixation between the spinous processes and CHA spacers (C4–6) using silk thread

mFDL was performed according to the method established by Kurokawa et al. [4] with the following modifications. During exposure of the laminae and spinous processes, the origins and terminations of the cervical semispinalis at the spinous processes were preserved. A 3-mm-wide groove was then made bilaterally at the lamina–facet junction line, paying attention to avoid resection of the inner cortex. After opening the split spinous processes in the double-door manner, the widened gap was grafted with a coralline hydroxyapatite (CHA) spacer (Bio-Osteon; Beijing YHJ Science and Trade Co., Ltd., Beijing, China) and fixed by silk thread (Fig. 2A–C).

### Evaluation and documentation

The patients’ demographic and clinical characteristics, including their body mass index (BMI), OPLL type, K-line status (+ or −), and follow-up duration, were recorded (Table 1). The following outcomes were evaluated: operative time, blood loss, neurological state (assessed by the Japanese Orthopaedic Association [JOA] score), and JOA score recovery rate ([postoperative JOA score − preoperative JOA score]/[17 − preoperative JOA score] × 100%) [11]. All surgery-related events that occurred within 30 days after the operation were defined as perioperative complications, among which C5 palsy was defined as deltoid and biceps motor weakness, paresthesia, or numbness in the distribution of the C5 nerve root without deterioration of myelopathic symptoms [12].

**Table 1:** Patients’ demographics

Variables	Total (*n* = 200)	mFDL (*n* = 69)	ODL (*n* = 131)	Statistics	*P* value
Age (years, $\overline{\mathrm{x} }$ ± *s*)	59.9 ± 9.1	62.0 ± 8.0	59.0 ± 9.0	*T* = 2.495	0.378
Gender				*χ*^2^ = 3.541	0.060
Male	144 (72.0%)	44 (63.8%)	100 (76.3%)		
Female	56 (28.0%)	25 (36.2%)	31 (23.7%)		
BMI (kg/m^2^, $\overline{\mathrm{x} }$ ± *s*)	25.5 ± 3.7	25.1 ± 3.5	25.6 ± 3.9	*T* = − 0.906	0.366
OPLL type				*χ*^2^ = 50.332	< 0.001
Continuous	71 (35.5%)	46 (66.7%)	25 (19.1%)		
Mixed	67 (33.5%)	18 (26.1%)	49 (37.4%)		
Segmental	51 (25.5%)	3 (4.3%)	48 (36.6%)		
Unclassified	11 (5.5%)	2 (2.9%)	9 (6.9%)		
K-line				*χ*^2^ = 0.295	0.587
Positive	155 (77.5%)	55 (79.7%)	100 (76.3%)		
Negative	45 (22.5%)	14 (20.3%)	31 (23.7%)		
*F*/*U* duration (month)	42 (36–54)	38 (36–42)	48 (35–70)	*U* = 6118.5	< 0.001
Operative time (min)	110 (90–150)	97 (90–120)	129 (92–166)	*U* = 6231.5	< 0.001
Blood loss (ml)	200 (100–400)	300 (200–400)	200 (25–400)	*U* = 3225.5	0.001
Pre-JOA score	11.3 (9.0,14.0)	9.0 (8.0,12.5)	12.0 (10.0,15.0)	*U* = 5915.5	< 0.001
Post-JOA score	15.0 (13.0–16.0)	15.0 (14.0–16.0)	15.0 (12.0–16.7)	*U* = 4521.5	0.996
JOA score’s RR	0.60 (0.05–0.82)	0.67 (0.50–0.75)	0.47 (0–0.89)	*U* = 4003.0	0.183

The preoperative JOA score, OPLL type, *F*/*U* duration, operative time, blood loss, postoperative JOA score, and JOA score RR are presented as median (interquartile range)

*mFDL* modified French-door laminoplasty, *ODL* open-door laminoplasty, *BMI* body mass index, *JOA* Japanese Orthopaedic Association, *OPLL* ossification of the posterior longitudinal ligament, *F/U* follow-up, *RR* recovery rate

### Statistical analysis

SPSS 22.0 (IBM Corp., Armonk, NY, USA) was used for the statistical analysis. The Kolmogorov–Smirnov test was performed to assess the normality of the distribution of continuous variables. If the distribution was normal, the results were described as mean ± standard deviation; otherwise, they were described as median (interquartile range). Classification variables were described as number and percentage. The independent-samples *t* test or Mann–Whitney U test was used to compare the differences in continuous variables between the two groups. Intergroup comparisons of categorical variables were made using Pearson’s Chi-square test or Fisher’s exact test. Bonferroni correction was used for multiple comparisons. The paired rank sum test was used to compare the differences in the JOA score in each center before and after treatment.

Using the JOA score classification established by Khan et al. [13], the patients were divided into those with mild (≥ 14), moderate (9–13), and severe (< 9) disease. Thus, in each group (mFDL and ODL), the patients were divided into two subgroups bounded by 14. Because the JOA score recovery rate showed a non-normal distribution, its 75% quantile was 0.82. Therefore, in each group (mFDL and ODL), the patients were divided into two subgroups bounded by 0.82. Because the operative time and blood loss also showed a skewed distribution, the clinical practice and characteristics of the data distribution were combined, and the operative time was then bounded by 90 min and blood loss by 300 mL. Binary logistic regression models adjusted for potential confounding factors were used to assess the association between the surgical methods and different outcomes. In accordance with convention, a *P* value of < 0.05 was considered statistically significant.

## Results

Two patients were not enrolled because of incomplete medical records. The demographics of the remaining 200 patients (144 men, 56 women) are summarized in Table 1. The patients’ age, sex, BMI, K-line status, postoperative JOA score, and JOA score recovery rate were not significantly different between the mFDL and ODL groups (all *P* > 0.05). However, the OPLL type, follow-up duration, operative time, blood loss, and preoperative JOA score were significantly different between the mFDL and ODL groups (all *P* < 0.05). Although the preoperative JOA score was different between the two groups, the postoperative JOA score and JOA score recovery rate were similar.

Compared with the preoperative JOA score, the last postoperative JOA score was significantly improved in both groups (15.0 [14.0–16.0] and 15.0 [12.0–16.7], respectively; *P* < 0.05) (Table 2). This finding indicates that both mFDL and ODL produced satisfactory outcomes in treating patients with C-OPLL.

**Table 2:** Comparison between preoperative and postoperative JOA scores in four institutions (A–D)

Variables	A (ODL)			B (ODL)			C (ODL)			D (mFDL)		
	Median (IQR)	*Z* value	*P* value	Median (IQR)	*Z* value	*P* value	Median (IQR)	*Z* value	*P* value	Median (IQR)	*Z* value	*P* value
Pre-JOA score	11.0 (9.0–13.0)	− 5.163	< 0.001	15.0 (12.0–16.0)	− 2.471	0.013	10.0 (8.0–11.5)	− 2.788	0.005	9.0 (8.0–12.5)	− 6.709	< 0.001
Post-JOA score	14.0 (12.0–16.0)			16.5 (12.0–17.0)			12.0 (11.0–15.5)			15.0 (14.0–16.0)		

*JOA* Japanese Orthopaedic Association, *ODL* open-door laminoplasty, *mFDL* modified French-door laminoplasty, *IQR* interquartile range

As shown in Table 3, a multivariate analysis was performed to compare the last JOA score and its recovery rate, operative time, and blood loss between the two groups. The binary logistic regression showed no significant difference in the postoperative JOA score (≥ 14; odds ratio [OR], 0.393; 95% confidence interval [CI] 0.147–1.050; *P* = 0.062). The JOA score recovery rate was ≥ 0.82 (OR, 1.844; 95% CI 0.743–4.576; *P* = 0.187), and the operative time was ≥ 90 min (OR, 0.956; 95% CI 0.375–2.439; *P* = 0.925). However, significantly more blood loss occurred in the mFDL group (OR, 0.342; 95% CI 0.162–0.720; *P* = 0.005).

**Table 3:** Multivariate analysis of association between type of laminoplasty and clinical outcomes

Variables*	*β*	SE	OR	95% CI	*P* value
post-JOA score	− 0.933	0.501	0.393	0.147–1.050	0.062
Operative time	− 0.045	0.478	0.956	0.375–2.439	0.925
Blood loss	− 1.073	0.380	0.342	0.162–0.720	0.005
JOA score’s RR	0.612	0.464	1.844	0.743–4.576	0.187

Independent variable: type of laminoplasty (groups: mFDL and ODL). The adjusted covariates for the JOA score and its RR were age, sex, BMI, preoperative JOA score, follow-up duration, OPLL type, and K-line. The adjusted covariates for operative time and blood loss were age, sex, BMI, preoperative JOA score, OPLL type, and K-line

*SE* standard error, *OR* odds ratio, *CI* confidence interval, *JOA* Japanese Orthopaedic Association, *RR* recovery rate

^*^Grouped by the variables below. Grouped by postoperative JOA score (bounded by ≥ 14), grouped by operative time (bounded by ≥ 90 min), grouped by blood loss (bounded by ≥ 300 mL), grouped by JOA score recovery rate (bounded by ≥ 0.82)

A stratified analysis was employed to explore the effect of the K-line and type of laminoplasty on the clinical outcomes of patients with C-OPLL (Tables 4 and 5). First, as shown in Table 4, the stratified analysis of the K-line indicated that regardless of the K-line status (+ or −), there were no significant differences in the postoperative JOA score or its recovery rate. More blood loss occurred in the K-line (+) patients treated by mFDL, but the blood loss in the K-line (−) patients was not significantly different between the two groups. Second, as shown in Table 5, the stratified analysis of the laminoplasty type indicated that regardless of whether mFDL or ODL was performed, there were no significant differences in the postoperative JOA score or its recovery rate, the operative time, or blood loss between the K-line (+) and (−) patients. This implies that K-line (−) patients can attain similarly effective outcomes as K-line (+) patients treated by mFDL or ODL.

**Table 4:** Stratified analysis of K-line for association between technical effectiveness and type of laminoplasty

Variables*	k-line (+)			K-line (−)		
	OR	95% CI	*P* value	OR	95% CI	*P* value
Post-operative JOA score	0.559	0.183–1.712	0.309	0.110	0.009–1.351	0.084
Operative time	0.770	0.265–2.238	0.631	0.875	0.059–12.962	0.922
Blood loss	0.244	0.100–0.597	0.002	0.659	0.112–3.878	0.644
JOA score’s RR	1.642	0.608–4.437	0.328	2.829	0.176–45.549	0.463

Stratification factors: K-line (+) and (−). Independent variables: type of laminoplasty (groups: mFDL and ODL). The adjusted covariates for the JOA score and its RR were age, sex, BMI, preoperative JOA score, follow-up duration, and OPLL type. The adjusted covariates for operative time and blood loss were age, sex, BMI, preoperative JOA score, and OPLL type

*OR* odds ratio, *CI* confidence interval, *JOA* Japanese Orthopaedic Association, *RR* recovery rate

^*^Grouped by the variables below. Outcome variables: grouped by postoperative JOA score (bounded by ≥ 14), grouped by operative time (bounded by ≥ 90 min), grouped by blood loss (bounded by ≥ 300 mL), grouped by JOA score recovery rate (bounded by ≥ 0.82)

**Table 5:** Stratified analysis of type of laminoplasty on association between effectiveness and K-line

Variables*	mFDL			ODL		
	OR	95% CI	*P* value	OR	95% CI	*P* value
Postoperative JOA score	1.799	0.371–8.732	0.466	2.329	0.836–6.486	0.106
Operative time	0.631	0.091–4.374	0.641	0.736	0.172–3.152	0.679
Blood loss	1.954	0.460–8.298	0.364	0.528	0.211–1.324	0.173
JOA score’s RR	2.864	0.284–28.897	0.372	1.376	0.507–3.739	0.531

*mFDL* modified French-door laminoplasty, *ODL* open-door laminoplasty, *OR* odds ratio, *CI* confidence interval, *JOA* Japanese Orthopaedic Association, *RR* recovery rate

^*^Grouped by the variables below. Outcome variables: grouped by postoperative JOA score (bounded by ≥ 14), grouped by operative time (bounded by ≥ 90 min), grouped by blood loss (bounded by ≥ 300 mL), grouped by JOA score recovery rate (bounded by 0.82). Stratification factors: type of laminoplasty (groups: mFDL and ODL). Independent variables: K-line (+) and (−). The adjusted covariates for the JOA score and its RR were age, sex, BMI, preoperative JOA score, follow-up duration, and OPLL type. The adjusted covariates for operative time and blood loss were age, sex, BMI, preoperative JOA score, and OPLL type

One patient in the ODL group developed an infection. C5 palsy occurred in seven patients in the ODL group and eight patients in the mFDL group (*χ*^2^ = 2.545, *P* = 0.142). No patients developed an epidural hematoma or dural tear. The operative time was 97 min (90–120 min) in the mFDL group and 129 min (92–166 min) in the ODL group (*P* < 0.001). The estimated blood loss was 300 mL (200–400 mL) in the mFDL group and 200 mL (25–400 mL) in the ODL group (*P* = 0.001).

## Discussion

Patients’ neurological function was satisfactorily improved after treatment with ODL or mFDL in this study. There were significant differences in the JOA score and the JOA score recovery rate between the preoperative and postoperative periods in both groups. Multivariate analysis confirmed that there was no significant difference in the JOA score, the postoperative JOA score recovery rate, or operation time between the ODL group and mFDL group, but significantly more blood loss occurred in the mFDL group. The stratified analysis demonstrated no significant difference in the JOA score, JOA score recovery rate, operation time, or blood loss between the K-line (+) and (−) patients undergoing ODL or mFDL, and there was no significant difference in neurological function of K-line (−) patients between the ODL and mFDL groups; however, more intraoperative bleeding occurred in the mFDL group. Therefore, the K-line status did not affect the recovery of neurological function of patients undergoing either laminoplasty technique. Finally, there was no significant difference in the incidence of perioperative complications between the two laminoplasty techniques.

The mFDL procedure adopted by our institution has several unique characteristics that differ from ODL. First, the enlarged spinal canal is symmetrical rather than irregular, as in ODL. Second, the CHA spacers located between the split spinous processes can induce the formation of an enclosed bony spinal canal, which prevents spinal cord compression caused by hyperplasia of scar tissue [14, 15]. Third, the CHA spacer can promote fusion with the split spinous processes as its osteoconductive feature. A retrospective study revealed continuous formation of the bony bridge in the interface between the spacers and spinous processes; the fusion rate reached 85%, which is a result similar to that provided by autografting [16, 17]. Fourth, the fusion between the CHA spacer and host bone is dependable and durable, and spacer dislodgement has rarely been reported. Face-to-face body fusion occurs, rather than the point-to-point body fusion in ODL, which only relies on limited numbers of small screws. Therefore, screw loosening is a common complication jeopardizing the recovery of neurological function after ODL [18]. Liu et al. [19] reported that screw back-out was noted in 16% of plates and in 5% of screws, and the authors proposed three factors leading to these complications: (1) the screw purchase was suboptimal because of a poor screw insertion trajectory, especially at the most cranial level; (2) screw retightening failed because of the imperfect design of the mini-plate; and (3) the screw was too small to obtain a dependable bony purchase. Compared with conventional FDL, the two main modifications described in this article are as follows. First, the C3 laminoplasty was replaced by laminectomy. As Takeuchi et al. [20] reported, the width of the semispinalis cervicis (SSC) at the C2 insertion is narrower than the CHA spacer, and the split spinous process and enlarged C3 lamina might therefore interfere with the course of the SSC in most cases. This tends to induce kyphotic change of the cervical alignment because the SSC is regarded as the most important muscle in the maintenance of cervical lordosis. Laminectomy will thus be in favor of preserving the normal function of the SSC. Moreover, the spinous process was sawed at its midpoint using a titanium saw and preparation of the bilateral gutters was replaced by simple laminectomy, which not only saved time but made the manipulation much safer to perform. Second, the spacer at the C3 and C7 levels were omitted. Too-dense application of spacers compromises the normal cervical range of motion, especially extension, which increases the risk of postoperative axial symptoms [15].

Which laminoplasty technique has greater effectiveness for the neurological recovery of patients with C-OPLL remains controversial. One meta-analysis showed that ODL resulted in a higher postoperative JOA score than FDL. Lee et al. [6] found that the JOA score recovery rate was significantly higher in ODL than FDL, and they attributed the better neurological improvement in ODL to greater enlargement of the spinal canal with ODL than FDL, providing more room for the spinal cord to recover its function. However, Koda et al. [21] and Chen et al. [22] reported no significant differences in the postoperative JOA score or JOA score recovery rate between the two groups. Wang et al. [23] demonstrated that both ODL and FDL could provide sufficient canal expansion to produce a dorsal spinal cord shift of > 3 mm, which was associated with a satisfactory outcome. However, excessive opening of the canal will cause various problems, such as C5 palsy and hypertrophy of epidural scar tissues [24, 25]. Our results are in accordance with those reported by Wang et al. [23], who reported that neurological improvement mainly relies on the degree of spinal canal enlargement instead of which laminoplasty technique is applied. Because both ODL and FDL can provide enough room for the spinal cord to recover, the surgical outcome with respect to neurological function is not substantially different between the two procedures.

Lee et al. [6] reported a non-significant tendency of more blood loss in the FDL than ODL group, which might have resulted from the need to drill more gutters in the FDL group. In the present study, the higher amount of blood loss in the mFDL group can likely be attributed to the performance of C3 laminectomy because this procedure required drilling at the border of the lamina and facet, where the congested venous plexus is usually located. Direct hemostasis was difficult to achieve by bipolar coagulation; thus, compression was required for hemostasis, unavoidably resulting in more blood loss. Although the operative time was significantly different between the ODL and mFDL groups in the univariate analysis, there was no significant difference in the multivariate analysis. This may have been due to the impact of potential covariates such as the BMI and the type of OPLL.

We also found no significant difference in the postoperative JOA score or JOA score recovery rate between the K-line (+) and K-line (−) groups treated by either ODL or mFDL. The K-line was proposed by Fujiyoshi et al. [26] and is used to evaluate both the cervical alignment and the size of OPLL. The authors proposed that K-line (−) patients tend to have a significantly lower recovery rate than K-line (+) patients following laminoplasty because posterior shifting of the spinal cord is inadequate. However, our results are consistent with those reported by Nagashima et al. [27], who found at the 2-year follow-up that the K-line had no impact on the neurological recovery of patients who underwent laminoplasty for C-OPLL. To our knowledge, application of the K-line in prognosis prediction has three limitations. (1) It can be difficult to clearly judge the OPLL and/or the midpoints of the spinal canal at C7 on a lateral radiograph compared with a computed tomography image, especially for overweight/obese and short patients, and this results in unavoidable interobserver error. (2) Because the K-line can be easily affected by patients’ cervical position when taking a radiograph, more attention is now being paid to the K-line in the flexed-neck position instead of the K-line (+)/(−) status in the neutral position [28]. Studies have demonstrated that a flexion-K-line (−) status is significantly associated with poorer functional recovery, which may be attributable to dysfunction of anterior horn cells induced by repetitive ventral compression of the spinal cord in the flexed-neck position [29–32]. (3) Some patients with C-OPLL develop kyphotic change after laminoplasty, making it difficult to predict whether the K-line will become negative following surgery, even in patients exhibiting a positive K-line preoperatively [33]. Loss of cervical lordosis after laminoplasty reportedly ranges from 7° to 12° [34, 35]. Thus, it is not appropriate to use the K-line status to predict the effectiveness of laminoplasty for patients with C-OPLL because the K-line is likely to be influenced by many factors.

This study has several limitations. First, it was a retrospective study, not a randomized controlled study, making potential bias inevitable. However, a multivariate analysis with adjustment for confounding factors and a stratified analysis of surgical methods and K-line characteristics were performed. Second, the mFDL procedures were performed in only one institution, whereas the ODL procedures were performed in the other three institutions. There were significantly fewer patients in the mFDL than ODL group, and this asymmetrical distribution of patients between the two groups may have led to bias. Third, this study only compared the JOA score and its recovery rate among different units to explore the effect of the K-line on the outcome. To make this study more comprehensive, other items should be addressed, such as radiographic evaluation findings and the occurrence and severity of axial symptoms. Fourth, the follow-up time was relatively short. Laminoplasty is an indirect decompressive procedure regardless of whether it is performed by mFDL or ODL. Various sequelae affecting the prognosis are not uncommon, such as progression of OPLL and loss of lordosis during follow-up [36]. Fifth, because this was a multicenter study involving several orthopedic spine surgeons or neurosurgeons from three countries in East Asia, bias in the distinction of operative techniques among these surgeons was inevitable. However, significant deviation was absent because all of the surgeons were from famous university hospitals in their own country and were well experienced in cervical laminoplasty. Finally, the sample size of this study was small and the hypothesis testing was insufficient. The results need to be further verified in a large-sample study. Despite these limitations, this was a multicenter study assessing the difference in effectiveness between mFDL and ODL as well as the effect of the K-line on the outcomes of patients who underwent different surgical procedures among three countries in East Asia that are considered to have the highest incidence rates of C-OPLL.

## Conclusion

This study has demonstrated that both ODL and mFDL can significantly improve neurological function after surgery in patients with C-OPLL. However, more blood loss occurs in mFDL than ODL. Laminoplasty may be an alternative treatment for K-line (−) patients because the surgical effectiveness is not significantly different between K-line (+) and (−) patients. Despite the fact that this study involved multiple centers among three countries, C-OPLL is an uncommon disease in the field of spinal surgery, and the sample size was therefore limited compared with investigations of other common spinal diseases. Therefore, our findings require further verification in a strictly designed large-sample study.

## Data Availability

The datasets generated and/or analyzed during the current study are not publicly available because the data were collected from four units in three countries; however, the data are available from the corresponding author on reasonable request.

## Abbreviations

ODL: Open-door laminoplasty
FDL: French-door laminoplasty
C-OPLL: Cervical ossification of the posterior longitudinal ligament
JOA: Japanese Orthopaedic Association
CHA: Coralline hydroxyapatite
BMI: Body mass index
SSC: Semispinalis cervicis
